# CONDUCTING A NEEDS ASSESSMENT TO INFORM AGE-FRIENDLY HEALTH SYSTEMS INITIATIVE FOR UNIVERSITY OF UTAH HEALTH

**DOI:** 10.1093/geroni/igad104.3245

**Published:** 2023-12-21

**Authors:** Ashley Cadiz, Rebekah Perkins, Timothy Farrell, Linda Edelman

**Affiliations:** University of Utah, Salt Lake City, Utah, United States; University of Utah, Salt Lake City, Utah, United States; University of Utah, Salt Lake City, Utah, United States; University of Utah, Salt Lake City, Utah, United States

## Abstract

Today, people live longer and more productive lives than ever. The number of adults aged 65 and older is projected to nearly double 98 million by 2060 to 24% of the U.S. population (Administration on Aging & Administration for Community Living, 2018). Increased life expectancy, coupled with the exponential growth of the older adult population will create more demand for healthcare professionals to meet the care needs of older adults living with chronic conditions. Chronic conditions can lead to complex care and increase the usage of healthcare systems (Axon & Kamel, 2021). The Age-Friendly Health Systems initiative provides specific evidenced-based best-practice interventions to all older adults in a health system setting (Fulmer et al., 2022). An Age-Friendly Health System is one in which every older adult’s care is guided by an essential set of evidence-based practices known as the 4Ms framework: What Matters, Medications, Mentation, and Mobility (Institute for Healthcare Improvement, 2022). This project developed and delivered a Needs Assessment to providers practicing in the U Health Ambulatory Care Clinics to identify baseline knowledge, gaps in knowledge, and current practices related to the AFHS 4Ms Framework. The Needs Assessment consists of using online surveys and semi-structured interviews. Significant findings were that: 1) U Health ambulatory care clinic providers have very little to no baseline knowledge of the AFHS 4Ms Framework, 2) U Health ambulatory care providers are already providing care that aligns with the 4Ms framework, 3) There are barriers and challenges to providing Age-Friendly care.

